# Effects of self-management interventions on self-care ability and quality of life in patients with urinary ostomy after bladder cancer: a systematic review and meta-analysis

**DOI:** 10.3389/fonc.2026.1864061

**Published:** 2026-07-27

**Authors:** Jiali Tian, Li Li, Lingli Xu, Ling Liu

**Affiliations:** 1 Department of Urology, West China Tianfu Hospital, Sichuan University, Chengdu, Sichuan, China; 2 Department of Urology, West China Hospital, Sichuan University, Chengdu, Sichuan, China

**Keywords:** bladder cancer, meta-analysis, quality of life, self-care ability, self-management, urinary ostomy

## Abstract

**Objective:**

To systematically assess the effects of structured self-management interventions on self-care ability, quality of life (QoL), anxiety, and stoma-related complications in bladder cancer patients with urinary ostomy, providing evidence to guide clinical practice and intervention design.

**Methods:**

A systematic search of CNKI, WanFang, VIP Database, CINAHL, Cochrane Library, PubMed, Embase, and Web of Science was conducted from January 1, 2016, to January 1, 2026. Randomized controlled trials evaluating self-management interventions in bladder cancer patients with urinary ostomy were included. Two reviewers independently performed study selection, data extraction, and quality assessment. Meta-analysis was conducted using Review Manager 5.4, and effect sizes were expressed as standardized mean difference (SMD) or risk ratio (RR) with 95% confidence intervals (95% CI). Heterogeneity was assessed using the I^2^ statistic.

**Results:**

A total of 27 RCTs involving 1,674 patients were included. Self-management interventions significantly improved self-care ability (SMD = 2.42, 95% CI: 1.63–3.22, *P* < 0.001), with substantial heterogeneity (I^2^ = 93.7%). QoL was also significantly improved (SMD = 1.44, 95% CI: 0.46–2.42, *P* < 0.01; I^2^ = 96%), with wide prediction intervals indicating variability across settings. Anxiety levels were significantly reduced (SMD = –1.12, 95% CI: –1.44 to –0.80, *P* < 0.001; I^2^ = 21.9%), and stoma-related complications were markedly decreased (RR = 0.24, 95% CI: 0.19–0.32, *P* < 0.001; I^2^ = 0%). Sensitivity analyses confirmed the robustness of the findings, and subgroup analyses suggested that intervention characteristics and follow-up duration contributed to heterogeneity.

**Conclusion:**

Structured self-management interventions improve self-care ability, QoL, psychological well-being, and stoma-related outcomes in bladder cancer patients with urinary ostomy. Integration into routine postoperative care is recommended, while future multicenter trials with standardized protocols and longer follow-up are needed to strengthen evidence.

**Systematic review registration:**

https://www.crd.york.ac.uk/PROSPERO/view/CRD420261424084, identifier CRD420261424084.

## Introduction

In 2020, bladder cancer was predicted to account for 213,000 fatalities and 573,000 new cases, making it the tenth most common cancer globally (1). Due to its high recurrence rate and the need for lifelong surveillance, bladder cancer imposes a substantial and sustained burden on patients, caregivers, and health care systems worldwide. There was significant geographic variation in its incidence, with the highest rates recorded in North America and Europe, and it is more common in men than in women (2). Muscle-invasive bladder cancer often requires radical surgery with urinary diversion, including ileal conduit, cutaneous ureterostomy, orthotopic neobladder, or continent urinary reservoirs (3). While these procedures are life-saving, they profoundly alter urinary function and daily life, and patients frequently experience physical discomfort, stoma-related complications, and psychological challenges including anxiety, depression, and social isolation (4). Effective postoperative adaptation is crucial for improving QoL and long-term outcomes.

Self-management interventions are structured approaches designed to help patients actively participate in their own care, improve self-care ability, and promote physical and psychological recovery (5). Unlike conventional postoperative education alone, self-management emphasizes patients’ active engagement, decision-making, and sustained behavioral change across the continuum of care, particularly after hospital discharge. These interventions commonly include educational strategies including preoperative stoma education, teach-back sessions, and informational manuals; skill training including hands-on stoma care training, workshops, and problem-solving exercises; psychosocial support including motivational interviewing, peer support groups, and nurse–family–peer collaborative models; and technology-assisted guidance including WeChat-based follow-up, digital monitoring, or telehealth support (6–8). Importantly, these components are often delivered in an integrated manner and adjusted according to different recovery stages, care settings, and individual patient needs. Some programs are guided by theoretical frameworks including Orem’s self-care theory, the Self-Transcendence Theory, or the Information-Motivation-Behavioral Skills Model (IMB model), and often combine multiple components—education, skills training, behavioral guidance, and psychosocial support—tailored to patient needs (9–11). Theory-driven interventions may enhance the consistency, reproducibility, and sustainability of self-management behaviors by providing a clear mechanism for behavior change. Evidence suggests that these interventions can enhance patients’ self-care ability, improve health-related QoL, reduce psychological distress including anxiety, and decrease the incidence of stoma-related complications (12).

Patients with urinary ostomy have been the subject of numerous randomized controlled studies (RCTs) that have examined self-management strategies. The majority are single-center trials that assess different intervention components, durations, and theoretical frameworks with small sample sizes, usually 20 to 60 participants per group (12). Existing RCTs generally report improvements in self-care ability and quality of life, reductions in anxiety, and a lower incidence of stoma-related complications (12, 13). However, the interventions and outcome measures vary widely, and long-term follow-up data are limited. In addition, the methodological quality of some trials is moderate, with concerns regarding blinding, allocation concealment, and sample representativeness. Determining the efficacy of self-management therapies in this patient population is challenging due to the uneven results produced by these restrictions.

A comprehensive synthesis of the available evidence is therefore needed to clarify the overall effectiveness of structured self-management interventions and to explore their potential clinical value. The purpose of this meta-analysis was to provide evidence to guide clinical practice and inform the design of future interventions by systematically evaluating the effects of structured self-management interventions, which include education, skills training, psychosocial support, and technology-assisted guidance, on the ability of bladder cancer patients with urinary ostomy to take care of themselves, their quality of life, stoma-related complications, and their anxiety.

## Materials and methods

### Literature search strategy

A comprehensive literature search was conducted in China National Knowledge Infrastructure (CNKI), WanFang Database, VIP Database, CINAHL, Cochrane Library, PubMed, Embase, and Web of Science. The time frame for the search was January 1, 2016, through January 1, 2026. The search approach focused on bladder cancer, urinary ostomy, and concepts linked to self-management by combining Medical Subject Headings (MeSH) phrases with free-text terms. Key search terms included bladder cancer (bladder cancer, urinary bladder neoplasms), urinary ostomy (urinary ostomy, urostomy, ileal conduit, urinary diversion), and self-management (self-management, self-care, self-efficacy, health education, stoma care). The PubMed search strategy was: (“bladder cancer”[MeSH Terms] OR “urinary bladder neoplasms”[MeSH Terms] OR “bladder cancer”[Title/Abstract]) AND (“urinary ostomy” OR “urostomy” OR “ileal conduit” OR “urinary diversion”) AND (“self-management” OR “self-care” OR “self-efficacy” OR “health education” OR “stoma care”). The complete search strategies for all databases are provided in **Supplementary Table 1**. To find more eligible studies, the reference lists of the included studies and pertinent reviews were manually checked. To reduce publication bias, gray literature was also searched. Clinical trial registries, including ClinicalTrials.gov and the WHO International Clinical Trials Registry Platform (ICTRP), were screened to identify potentially unpublished or ongoing studies. This review was registered in PROSPERO (CRD420261424084). The reporting of this systematic review and meta-analysis followed the PRISMA 2020 guidelines (14).

### Eligibility criteria

Studies were included if they met the following criteria: (1) participants were adult bladder cancer patients who underwent urinary ostomy; (2) in the experimental group, self-management interventions were defined as interventions designed to support individuals in managing their health condition on a daily basis through education (providing knowledge about the consequences and management of their disease) and/or training (developing skills related to daily living, goal setting, and problem solving); the control group received routine care, standard education, or no additional intervention; (3) the study design was an RCT; and (4) outcomes included self-care ability, QoL, stoma-related complications, and anxiety. Studies that were review articles, had non-extractable or missing data, were conference abstracts or did not have full-text access, were duplicate publications, or were published in languages other than English or Chinese were not included.

### Study selection and data extraction

Two researchers with formal training in evidence-based nursing independently conducted study selection and data extraction. All retrieved records were imported into EndNote X9.2 for duplicate removal. After excluding irrelevant studies based on the titles and abstracts, the full-text review of any potentially suitable papers was conducted in accordance with the predetermined inclusion and exclusion criteria. Discussions were used to settle any disputes, and a third researcher—the third author—was consulted if agreement could not be reached. Author details, publication year, country, study design, participant characteristics, sample size, duration and frequency of interventions, format, theoretical framework, specifics of interventions in the experimental and control groups, and outcome measures were among the data that were independently extracted using a standardized data extraction form. When missing or incomplete data were encountered, attempts were made to contact the corresponding authors for clarification or additional information. If the required data could not be obtained, the study was included in the qualitative synthesis only, and was excluded from quantitative pooling for the affected outcomes.

### Assessment of certainty of evidence

The certainty of evidence for each outcome was assessed independently by two reviewers using the Grading of Recommendations Assessment, Development and Evaluation (GRADE) framework (15). Evidence from randomized controlled trials was initially rated as high certainty and was downgraded by one or two levels based on five domains: risk of bias, inconsistency, indirectness, imprecision, and publication bias. Summary of findings tables were generated using GRADEpro GDT software (McMaster University, 2020). Disagreements were resolved through discussion or consultation with a third reviewer.

### Quality assessment

The Cochrane Risk of Bias 2.0 (RoB 2.0) tool was used by two researchers to independently evaluate the methodological quality of the included randomized controlled trials. The RoB 2.0 tool assesses five domains: randomization process, deviations from intended interventions, missing outcome data, measurement of the outcome, and selection of the reported result (16). The risk of bias was rated as low, some concerns, or high for each domain. Studies were rated as having a low risk of bias, some concerns, or a high risk of bias according to the overall bias judgment. A third researcher was consulted or discussed with in order to settle any disagreements on quality assessment.

### Statistical analysis

Meta-analysis was conducted using Review Manager (RevMan) version 5.4. All pooled outcome measures were continuous variables, and effect sizes were expressed as mean difference (MD) or standardized mean difference (SMD) with corresponding 95% confidence intervals (95% CI). Statistical heterogeneity was assessed using the chi-square test and the I^2^ statistic. When *P* > 0.10 and I^2^ < 50%, heterogeneity was considered low and a fixed-effects model was applied; when *P* ≤ 0.10 and I^2^ ≥ 50%, substantial heterogeneity was assumed, potential sources of heterogeneity were explored, and if heterogeneity could not be sufficiently reduced, a random-effects model was employed. Clinical and methodological heterogeneity were further examined through sensitivity analyses, subgroup analyses, or descriptive analysis when meta-analysis was not appropriate. Publication bias was assessed using funnel plots and Egger’s regression test. All statistical tests were two-sided, and a *P* value < 0.05 was considered statistically significant.

## Results

### Literature search and study selection

**Figure 1:**
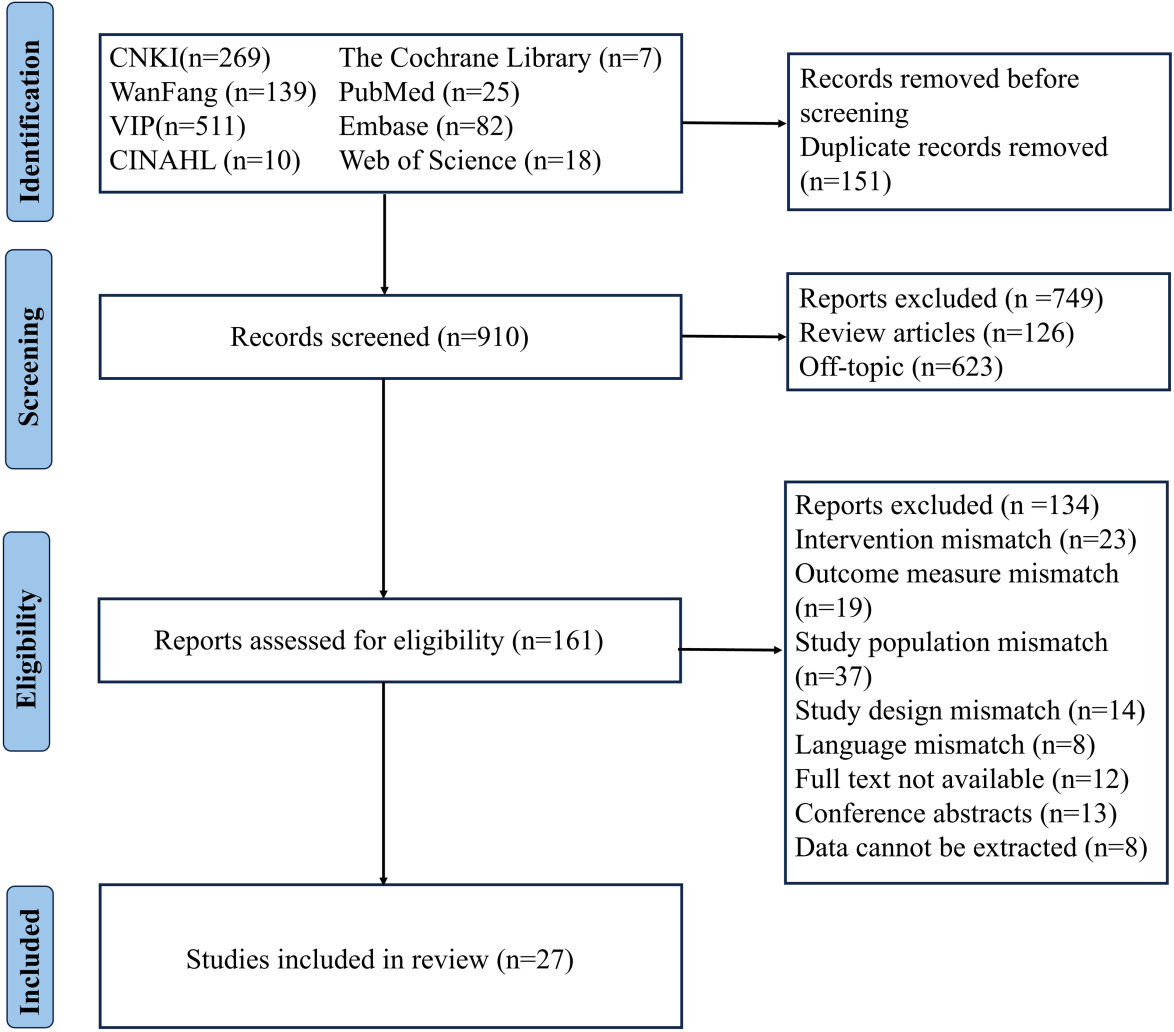
PRISMA flow diagram of the study selection process. This image illustrates the process of identifying and selecting studies for the systematic review and meta-analysis on self-management interventions for bladder cancer patients with urinary ostomy.

A comprehensive search of eight databases from January 2016 to January 2026 initially identified 1,061 records. After removing 151 duplicates, 910 articles underwent title/abstract screening. This led to the exclusion of 749 records, primarily review articles (n=126) or off-topic studies (n=623). Following full-text evaluation of the remaining 161 papers, 134 articles were excluded, primarily because of mismatched research populations (n = 37), interventions (n = 23), or results (n = 19). Ultimately, 27 studies were included in the review after fulfilling all eligibility requirements. The PRISMA flow diagram (**Figure 1**) describes the selection procedure in detail, and **Table 1** provides a summary of the fundamental traits of the selected research.

**Table 1:** Basic characteristics of included randomized controlled trials.

Author (Year)	Country	Sample size (T/C)	Experimental group	Control group	Duration/Follow-up	Outcomes
(25)	China	43/43	Nurse-family-peer collaborative self-management	Routine nursing care	Not reported	Self-care ability; Anxiety (HAMA)
(26)	China	50/50	Self-transcendence theory guided self-management program	Routine nursing care	4 weeks	Self-care ability (ESCA); Stoma-related complications
(27)	China	30/30	Teach-back and WeChat-based self-management guidance	Routine nursing care (verbal instruction + videos)	1 week	Self-care ability; QoL (SF-36); Anxiety (SAS)
(28)	China	35/34	ADOPT nursing–based self-management	Routine nursing care	3 months	Self-care ability; Stoma-related complications
(29)	China	46/46	Motivational interviewing–based self-management	Routine nursing care	1 month	Self-care ability (ESCA); QoL (GQOL-74); Stoma-related complications
(30)	China	40/40	Feedback-based self-management education	Routine health education	3 months	Self-care ability (Kristensen scale); Stoma-related complications
(31)	China	35/35	Solution-focused self-management program	Traditional nursing care	Post-intervention assessment	Self-care ability; QoL (SF-36); Stoma-related complications
(32)	China	45/45	Multidimensional self-management intervention	Routine nursing intervention	Post-intervention assessment	Self-care ability (Kristensen scale, ESCA); QoL (WHOQOL-100); Anxiety (HAMA)
(33)	China	38/38	TCM emotional nursing and clinical pathway self-management	Routine nursing care under CNP	2 months	Self-care ability; QoL (GQOLI-74)
(34)	China	50/50	IMB model–based self-management	Routine nursing care	6 months	Self-care ability; QoL (QLQ-C30); Stoma-related complications
(35)	China	40/41	Orem self-care model–based self-management	Routine nursing care	3 months	QoL (FACT-G); Anxiety (HADS); Stoma-related complications
(36)	China	41/41	Continuity of care and staged feedback self-management method	Continuity of care alone	3 months	Self-care ability (ESCA); QoL
(37)	China	46/46	Informatized continuity of care self-management	Routine follow-up care	1 month	Self-care ability (ESCA); Stoma-related complications
(38)	China	20/20	Self-care instruction and skills training	Routine nursing care	6 months	Stoma-related complications
(39)	China	39/39	Whole-process self-management nursing	Routine nursing care	Not reported	Anxiety and Depression (SAS/SDS); QoL (SF-36)
(40)	China	44/44	Detail-oriented self-management nursing	Routine nursing care	6 months	Self-care ability (ESCA); QoL (FACT-BL); Stoma-related complications
(41)	China	43/43	Structured continuity of care self-management	Routine nursing care and follow-up	12 months	Self-care ability (ESCA); QoL (QOL-RT)
(42)	China	50/50	Self-management support guided by Orem’s self-care theory	Routine nursing care	12 weeks	Self-care ability; QoL (SF-36); Stoma-related complications
(43)	China	45/45	Self-efficacy club self-management	Routine continuity of care	6 months	Self-care ability (ESCA); QoL (QLQ-C30)
(44)	China	23/23	Comprehensive self-management nursing	Routine nursing care	1 month	Self-care ability
(45)	China	26/26	Solution-focused self-management	Routine nursing care	3 months	QoL (WHOQOL-100); Stoma-related complications
(46)	Denmark	44/46	Preoperative stoma self-management education	No preoperative education	Up to 12 months postoperatively	Self-care skills (UES)
(47)	China	47/47	Comprehensive self-management nursing	Routine nursing care	1 month	Self-care ability
(48)	China	50/50	Health Belief Model–based self-management	Routine health education	8 weeks	Self-care ability (ESCA); QoL (COH-QOL-OQ); Stoma-related complications
(49)	China	45/45	Comprehensive self-management nursing	Routine nursing care	Not reported	Self-care ability (ESCA); QoL (SF-36)
(50)	China	63/63	Comprehensive self-management nursing	Routine perioperative nursing care	4 weeks	QoL (QLQ-C30)
(51)	China	32/34	Comprehensive self-management nursing	Routine nursing care	1 month	Self-care ability

T/C indicates the sample size of the intervention (treatment) group and control group. TCM, traditional Chinese medicine; CNP, clinical nursing pathway; IMB, Information-Motivation-Behavioral Skills; SDS, Self-Rating Depression Scale; RCT, randomized controlled trial; ESCA, Exercise of Self-Care Agency scale; QoL, quality of life; UES, Utrecht Stoma Education Scale; HAMA, Hamilton Anxiety Rating Scale; SAS, Self-Rating Anxiety Scale; HADS, Hospital Anxiety and Depression Scale.

### Risk of bias assessment

**Figure 2:**
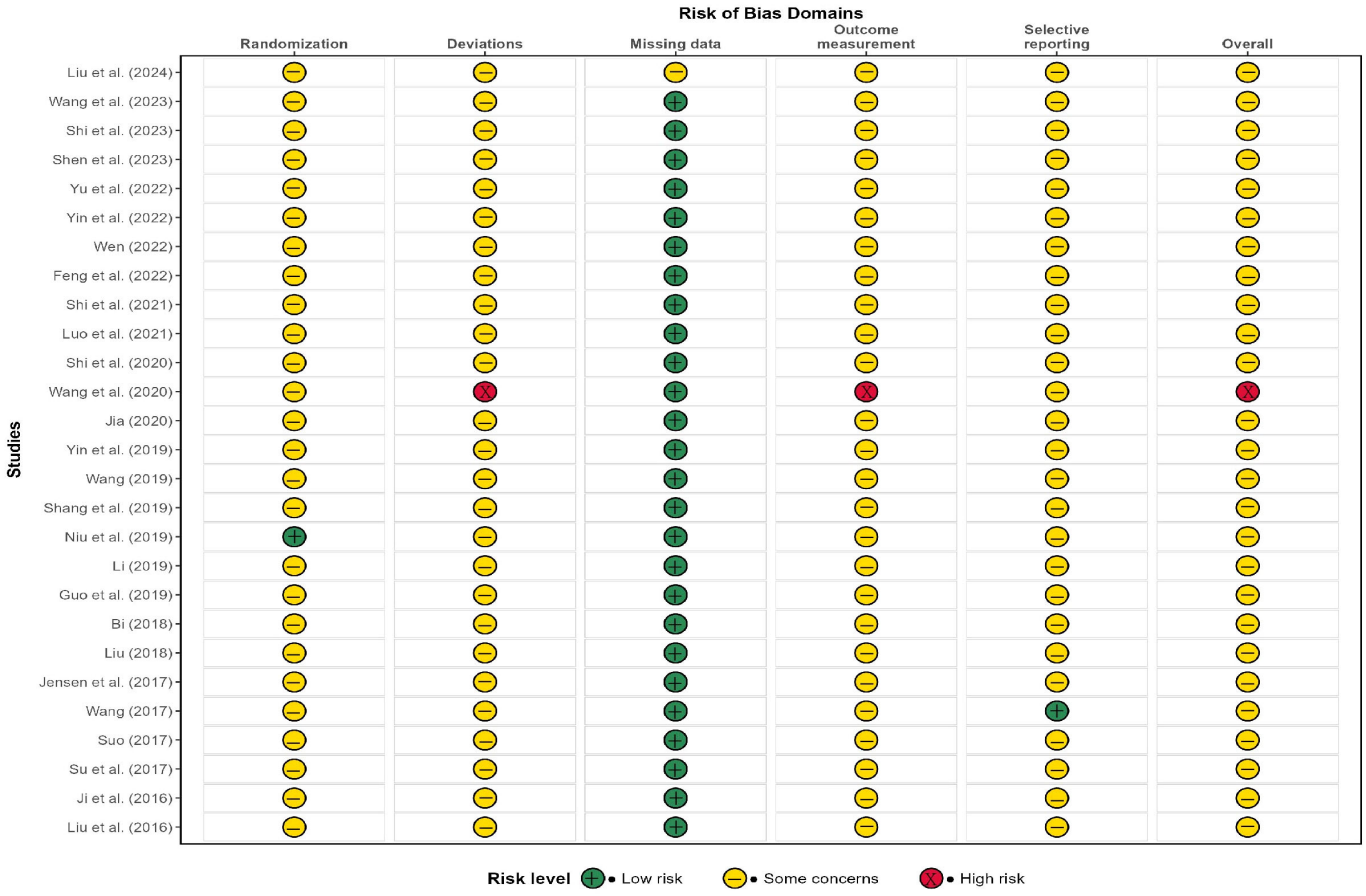
Risk of bias summary of included studies. Summary of the risk of bias assessment for each included study across five methodological domains: randomization process, deviations from intended interventions, missing outcome data, measurement of the outcome, and selection of the reported result, together with the overall risk of bias. Green (low risk), yellow (some worries), and red (high danger) are the colors used to represent different risk categories.

**Figure 3:**
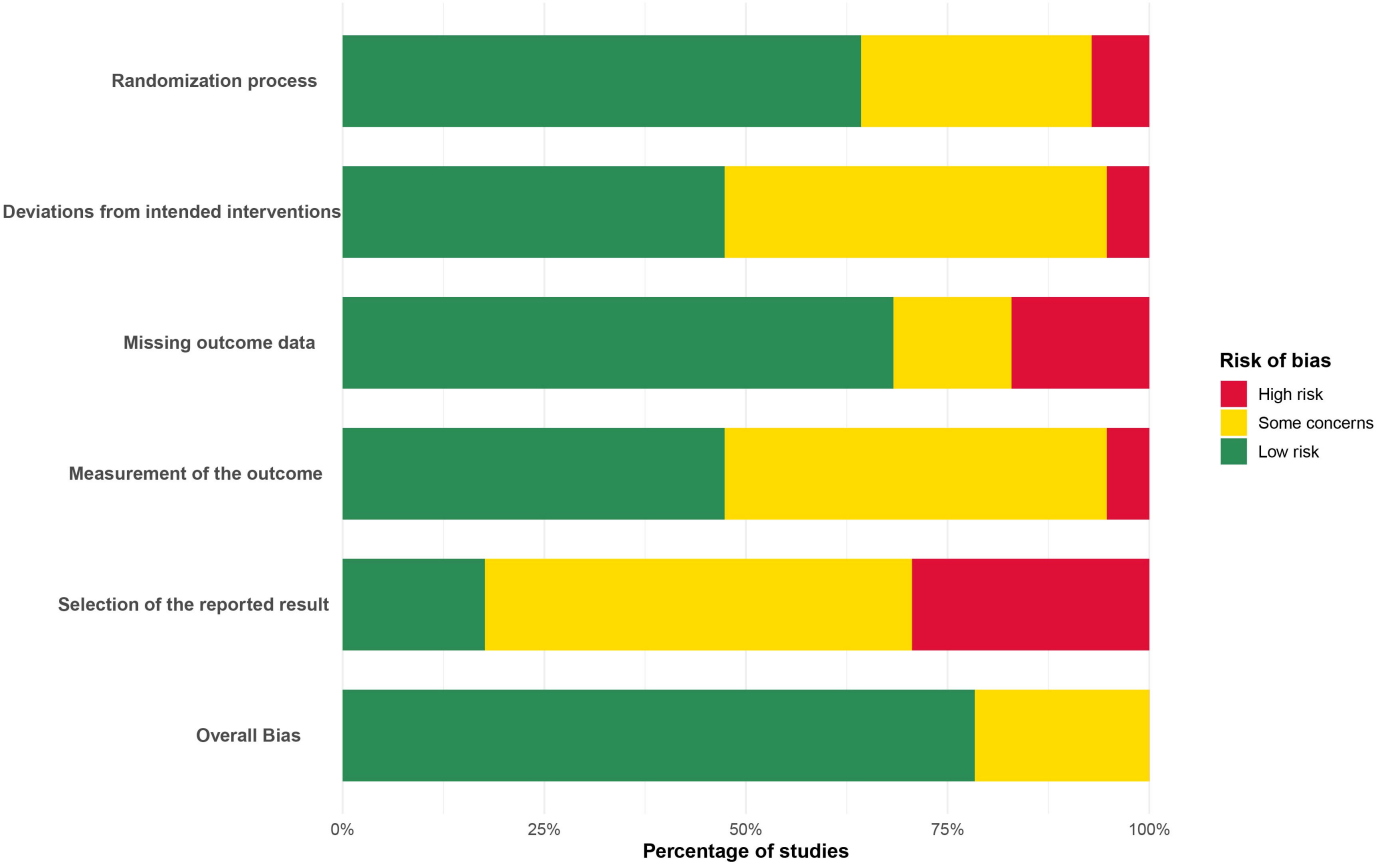
Risk of bias distribution across domains. Distribution of risk-of-bias judgments across studies for each domain and overall assessment. Bars represent the percentage of studies rated as low risk (green), some concerns (yellow), or high risk (red).

The Cochrane Risk of Bias 2.0 (RoB 2.0) tool was used to evaluate the methodological quality of the 27 included randomized controlled trials; comprehensive domain-level assessments are displayed in **Figure 2**, and the overall summary is displayed in **Figure 3**. One study (Wang et al., 2020) was rated as having a high overall risk of bias, while 26 other papers were deemed to have some concerns regarding risk of bias. Concerns related to the randomization process and deviations from intended interventions were common, primarily due to insufficient reporting of sequence generation, allocation concealment, and blinding procedures; Wang et al. (2020) was rated as high risk in both domains. The majority of studies were deemed to have a low risk of bias regarding missing outcome data, suggesting that outcome reporting was generally comprehensive. Since outcomes were patient-reported and assessor blinding was uncertain, the majority of trials in the measurement of outcomes area were rated as having some issues; once more, Wang et al. (2020) was deemed to be high risk. Due to the absence of publicly accessible procedures or trial registrations, most studies were deemed to have some concerns with selective reporting.

### Certainty of evidence

The certainty of evidence for all outcomes was assessed using the GRADE framework (**Supplementary Table 2**). Among the four outcome comparisons evaluated, one comparison was rated as moderate-quality evidence, and three comparisons were rated as low-quality evidence. No comparisons were rated as high or very low quality.

### Effects of self-management interventions on self-care ability

**Figure 4:**
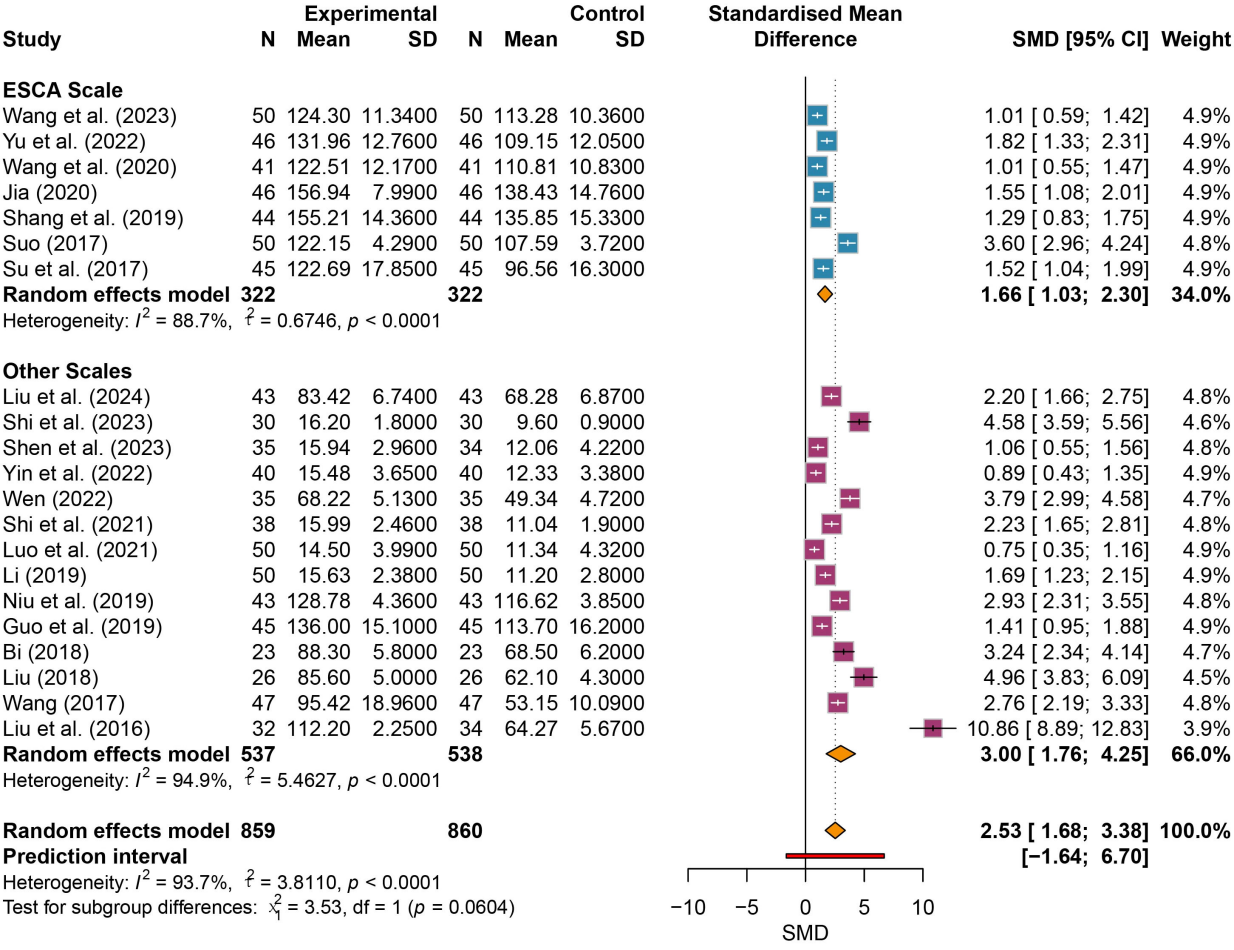
Forest plot of the meta-analysis on self-care ability. This image presents the pooled effect of self-management interventions on self-care ability in bladder cancer patients with a urinary ostomy.

Self-management interventions significantly increased self-care skill when compared to routine care, according to a pooled analysis of 20 studies with 1,257 participants. The overall effect size was large (SMD = 2.42, 95% CI: 1.63–3.22; *P* < 0.001; **Figure 4**). Studies showed significant heterogeneity (I^2^ = 93.7%). Subgroup analyses were carried out based on the measuring tools utilized in order to investigate possible sources of heterogeneity. Self-care ability was significantly improved by studies using the Exercise of Self-Care Agency (ESCA) scale (7 studies) (SMD = 1.66, 95% CI: 1.03–2.30; I^2^ = 88.7%), and by studies using other self-care-related scales (13 studies) (SMD = 3.00, 95% CI: 1.76–4.25; I^2^ = 94.9%).

**Figure 5:**
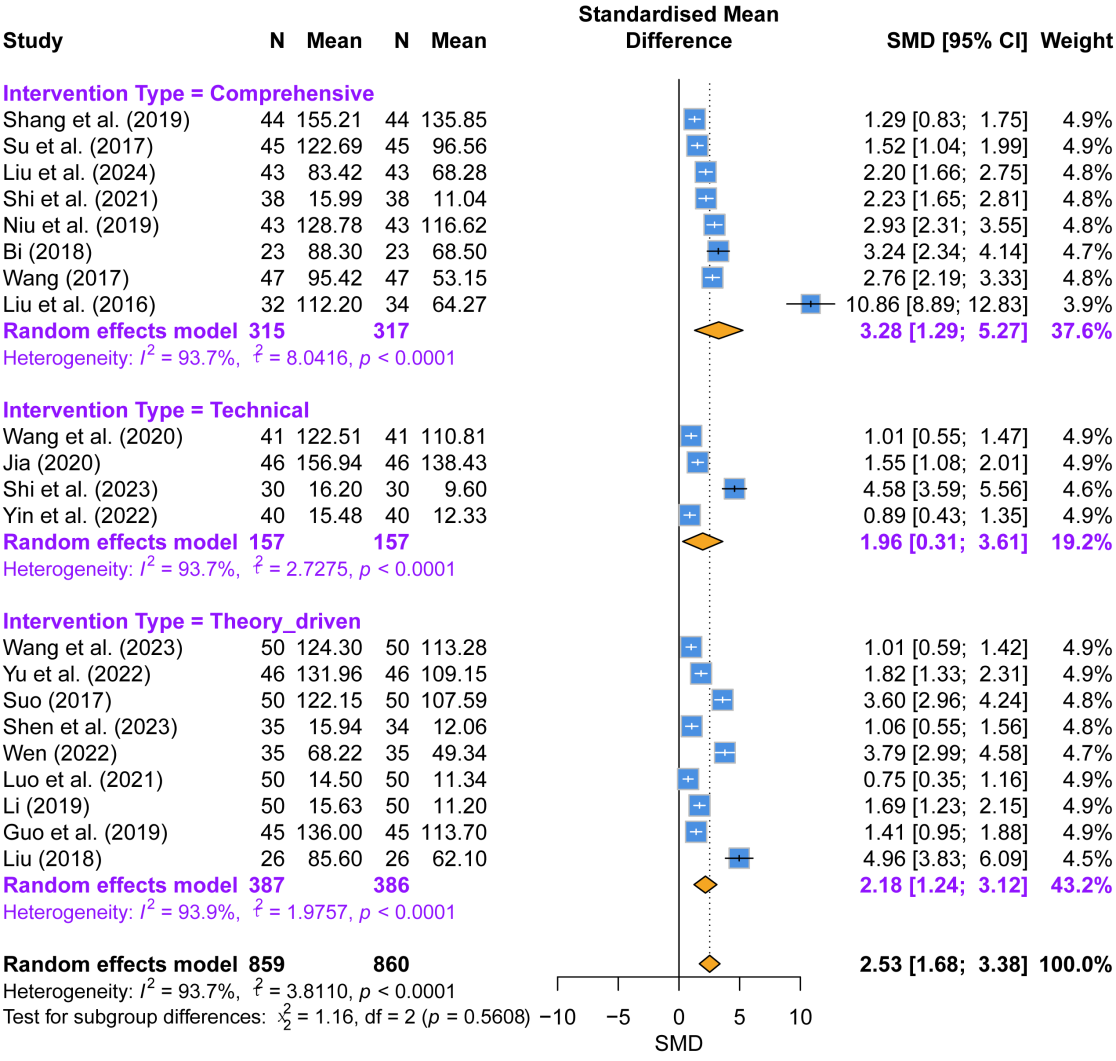
Forest plot of subgroup analysis on self-care ability by intervention type. This image presents the subgroup analysis of self-management interventions on self-care ability stratified by intervention type (comprehensive, theory-driven, and technical assistance interventions).

**Figure 6:**
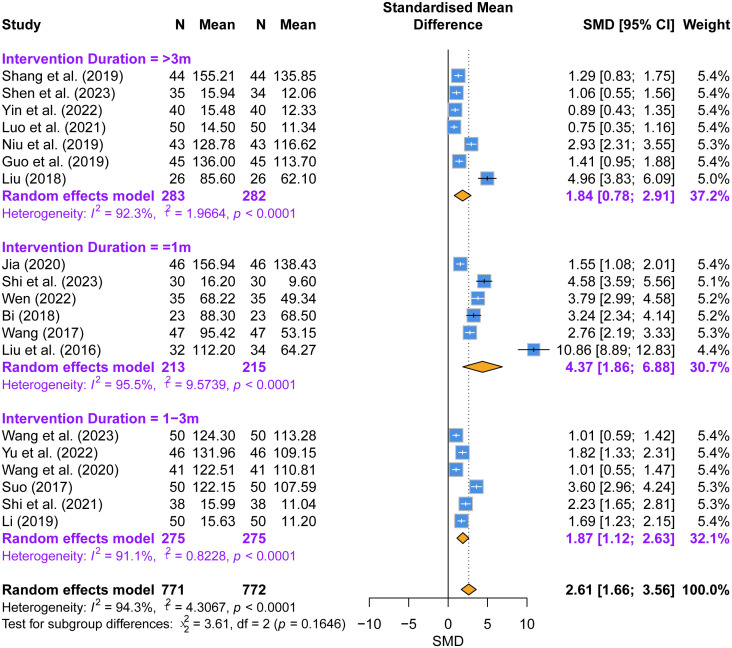
Forest plot of subgroup analysis on self-care ability by intervention duration. This image presents the subgroup analysis of self-management interventions on self-care ability stratified by intervention duration (≤1 month, 1–3 months, and >3 months).

**Figure 7:**
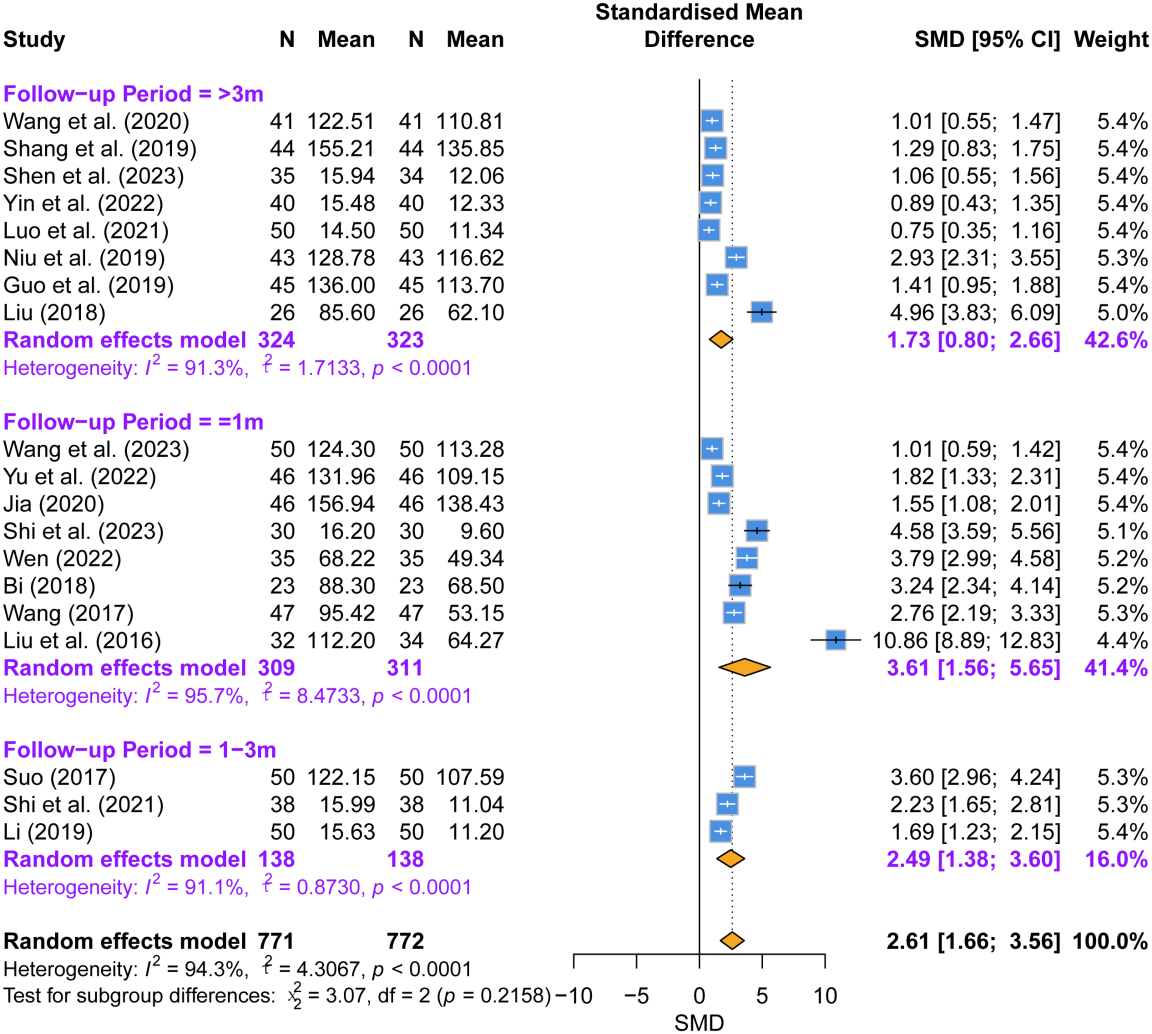
Forest plot of subgroup analysis on self-care ability by follow-up period. This image presents the subgroup analysis of self-management interventions on self-care ability stratified by follow-up period (≤1 month, 1–3 months, and >3 months).

To further explore the sources of heterogeneity, additional subgroup analyses were conducted based on intervention type, intervention duration, and follow-up period. Subgroup analysis by intervention type revealed that comprehensive interventions yielded the largest effect (SMD = 3.28, 95% CI: 1.29–5.27), followed by theory-driven (SMD = 2.18, 95% CI: 1.24–3.12) and technical assistance interventions (SMD = 1.96, 95% CI: 0.31–3.61; **Figure 5**). Subgroup analyses by intervention duration and follow-up period showed that studies with shorter durations (≤1 month) and shorter follow-ups (≤1 month) reported substantially larger effects (SMD = 4.37 and 3.61, respectively), whereas longer durations (>3 months) and longer follow-ups (>3 months) yielded more conservative estimates (SMD = 1.84 and 1.73, respectively; **Figures 6**, **7**).

**Figure 8:**
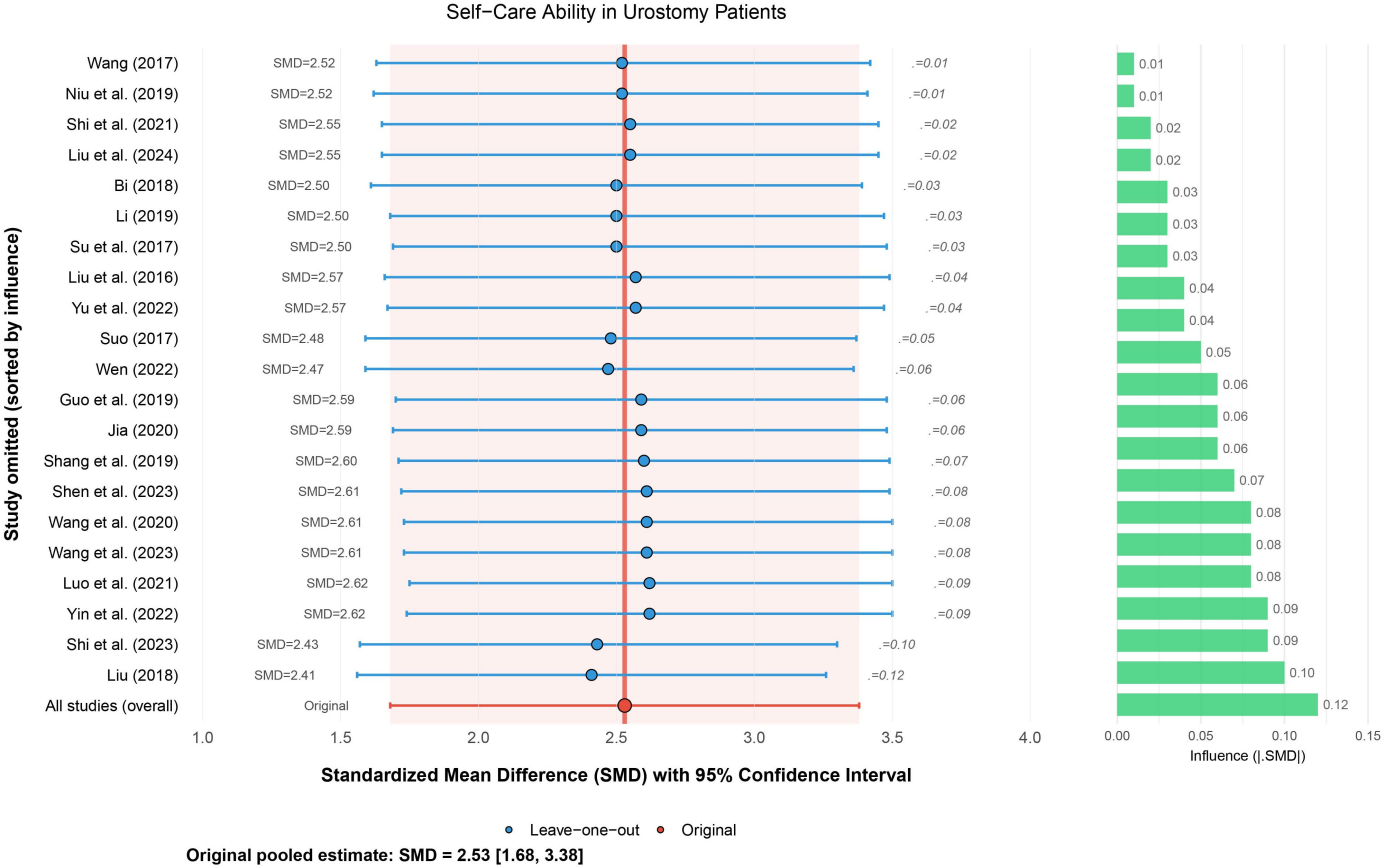
Leave-one-out sensitivity analysis for self-care ability. This image presents the results of a sensitivity analysis assessing the influence of individual studies on the pooled SMD for self-care ability.

To assess the robustness of the pooled effect estimate for self-care ability, a leave-one-out sensitivity analysis was performed (overall SMD = 2.42, 95% CI: 1.63 to 3.22; **Figure 8**). The results demonstrated that sequentially excluding any single study did not substantially alter the overall conclusion. The recalculated SMD estimates remained highly consistent, ranging from 2.41 to 2.62, with all 95% confidence intervals excluding zero and maintaining statistical significance. Specifically, the most influential exclusion (Liu et al., 2018) yielded an SMD of 2.41, while the least influential exclusions (e.g., Yu et al., 2022; Liu et al., 2016) yielded an SMD of 2.57.

**Figure 9:**
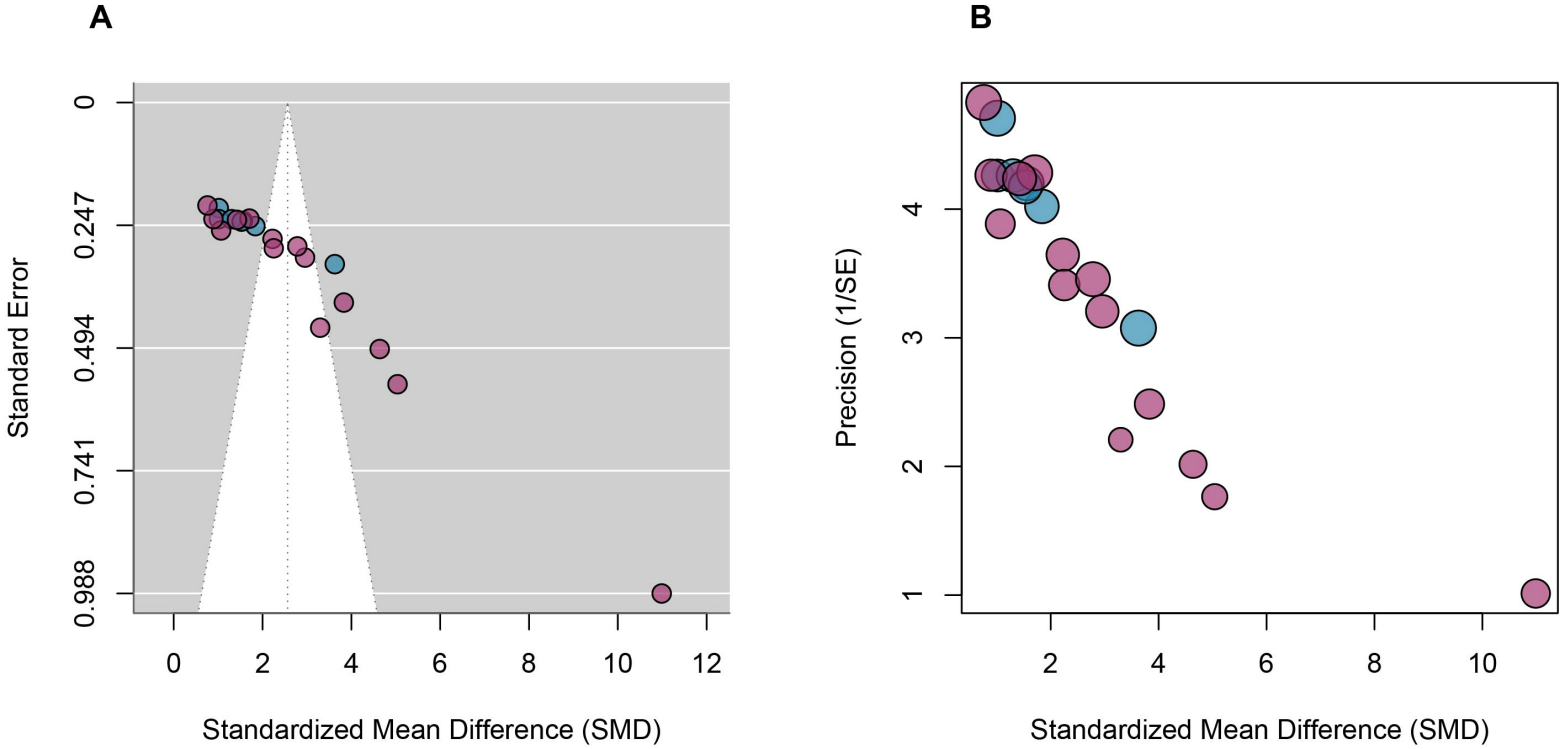
Assessment of publication bias using funnel and precision plots. **(A)** Funnel plot depicting the relationship between the standard error and SMD for each included study. **(B)** Precision plot (precision = 1/standard error) illustrating the association between study precision and effect size.

Publication bias was assessed using funnel plots and Egger’s regression test (**Figure 9**). Visual inspection of the funnel plot revealed significant asymmetry, with most studies clustered on the left near the pooled estimate and one outlier on the right showing a large effect size and low precision. Egger’s test confirmed significant publication bias (*P* < 0.001). The precision plot showed that studies with lower precision tended to report larger effects.

### Effects of self-management interventions on QoL

**Figure 10:**
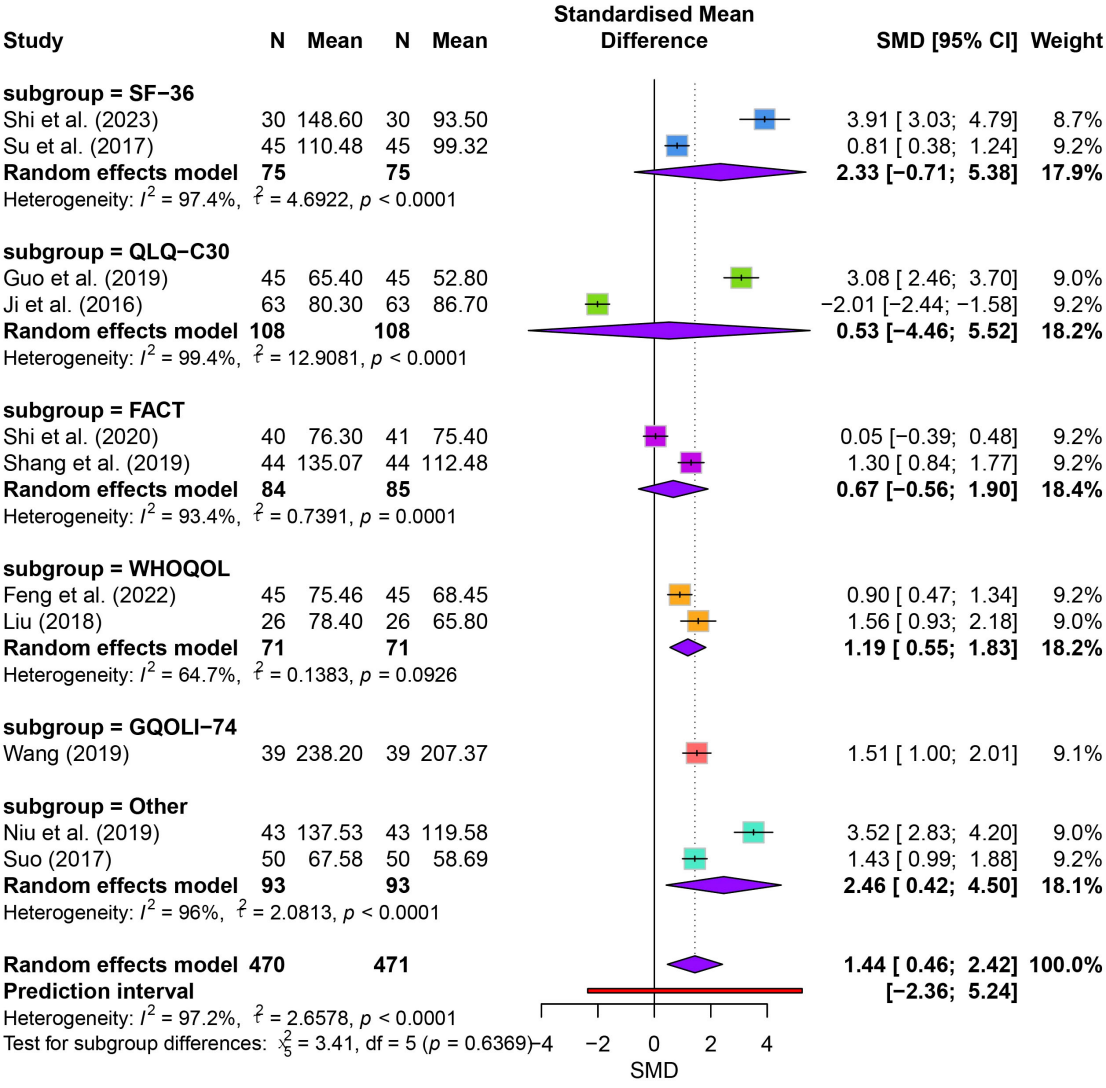
Forest plot of the meta-analysis on QoL. This image displays the pooled effect of self-management interventions on QoL in bladder cancer patients with a urinary ostomy.

In a pooled analysis of 10 research with 941 individuals, the impact of self-management treatments on quality of life was assessed. Given the substantial heterogeneity observed (I^2^ = 96%), a random-effects model was applied. The overall analysis showed a statistically significant improvement in QoL favoring the intervention group (SMD = 1.44, 95% CI: 0.46–2.42; *P* < 0.01; **Figure 10**); however, the wide prediction interval (–2.36 to 5.24) indicates considerable variability in the true effect across different settings. Subgroup analyses stratified by QoL measurement instruments demonstrated marked variation in effect estimates, with larger effects observed in studies using SF-36 (SMD = 3.91, 95% CI: 3.03–4.79) and QLQ-C30 (SMD = 3.08, 95% CI: 2.46–3.70), moderate effects in those using WHOQOL-100 (SMD = 0.90, 95% CI: 0.47–1.34), and a non-significant effect in studies employing the FACT series (SMD = 0.05, 95% CI: –0.39 to 0.48). Single-study subgroups using GQOLI-74 (SMD = 1.51, 95% CI: 1.00–2.01) and other QoL scales (SMD = 3.52, 95% CI: 2.83–4.20) also suggested favorable effects.

**Figure 11:**
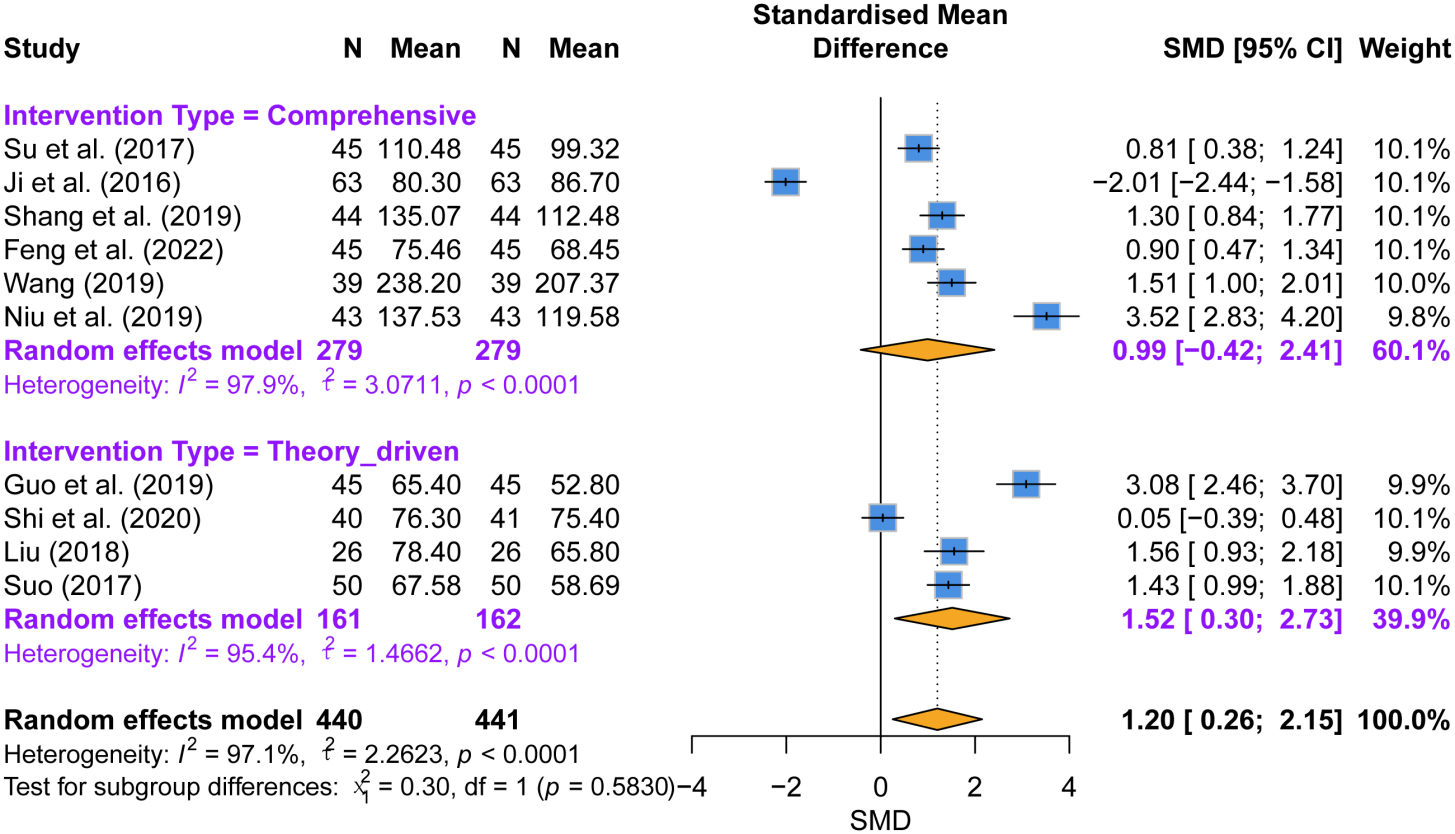
Forest plot of subgroup analysis on quality of life by intervention type. This image presents the subgroup analysis of self-management interventions on quality of life stratified by intervention type (comprehensive and theory-driven interventions).

**Figure 12:**
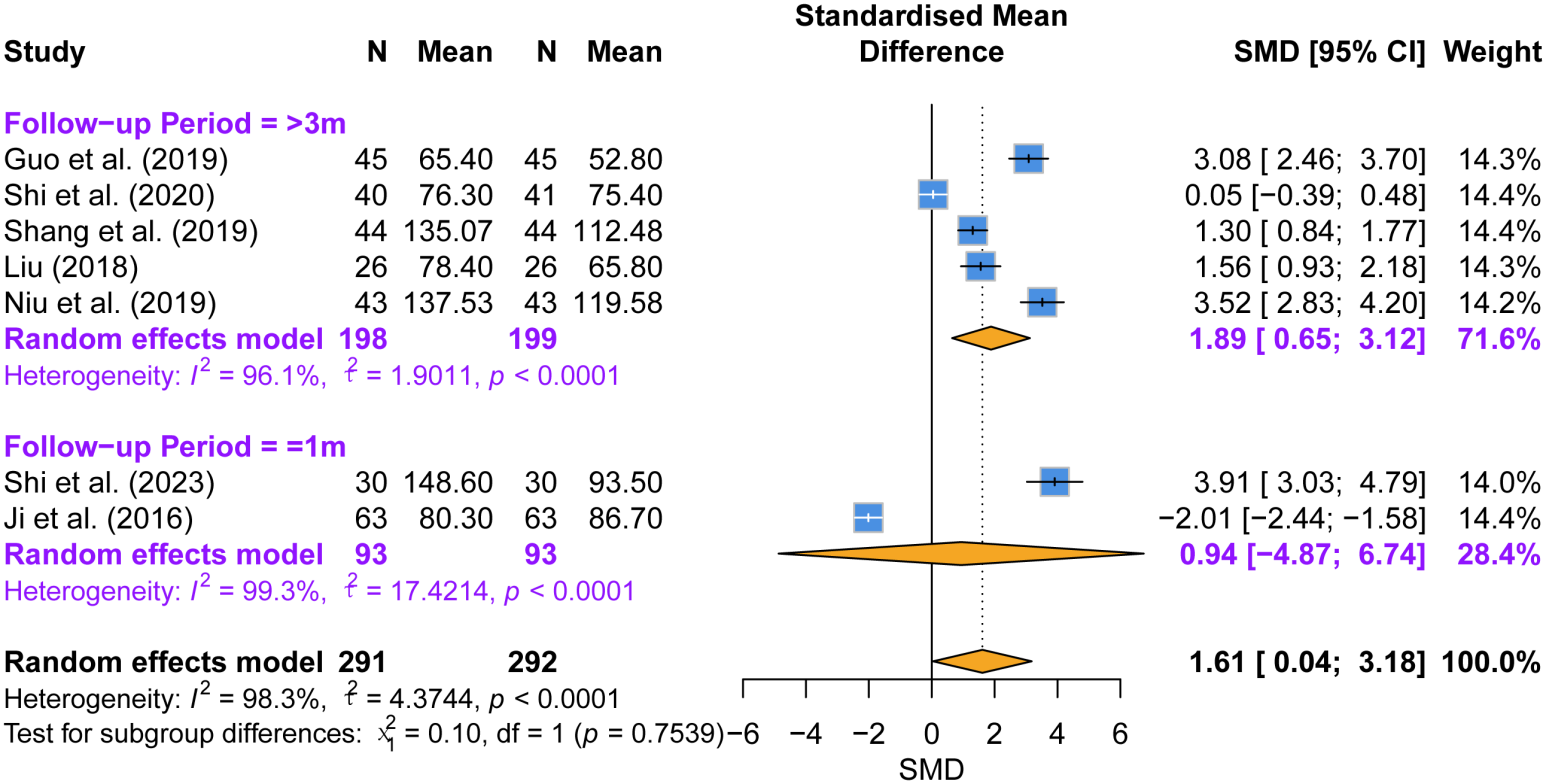
Forest plot of subgroup analysis on quality of life by follow-up period. This image presents the subgroup analysis of self-management interventions on quality of life stratified by follow-up period (≤1 month and >3 months).

To further investigate potential sources of heterogeneity, additional subgroup analyses were performed based on intervention type and follow-up period. Subgroup analysis by intervention type showed that theory-driven interventions yielded larger effects (SMD = 1.52, 95% CI: 0.30–2.73) compared with comprehensive interventions (SMD = 0.99, 95% CI: –0.42 to 2.41; **Figure 11**). Subgroup analysis by follow-up period demonstrated that studies with longer follow-up (>3 months) reported larger effects (SMD = 1.89, 95% CI: 0.65–3.12) than those with shorter follow-up (≤1 month; SMD = 0.94, 95% CI: –4.87 to 6.74; **Figure 12**).

### Effects of self-management interventions on stoma-related complications

**Figure 13:**
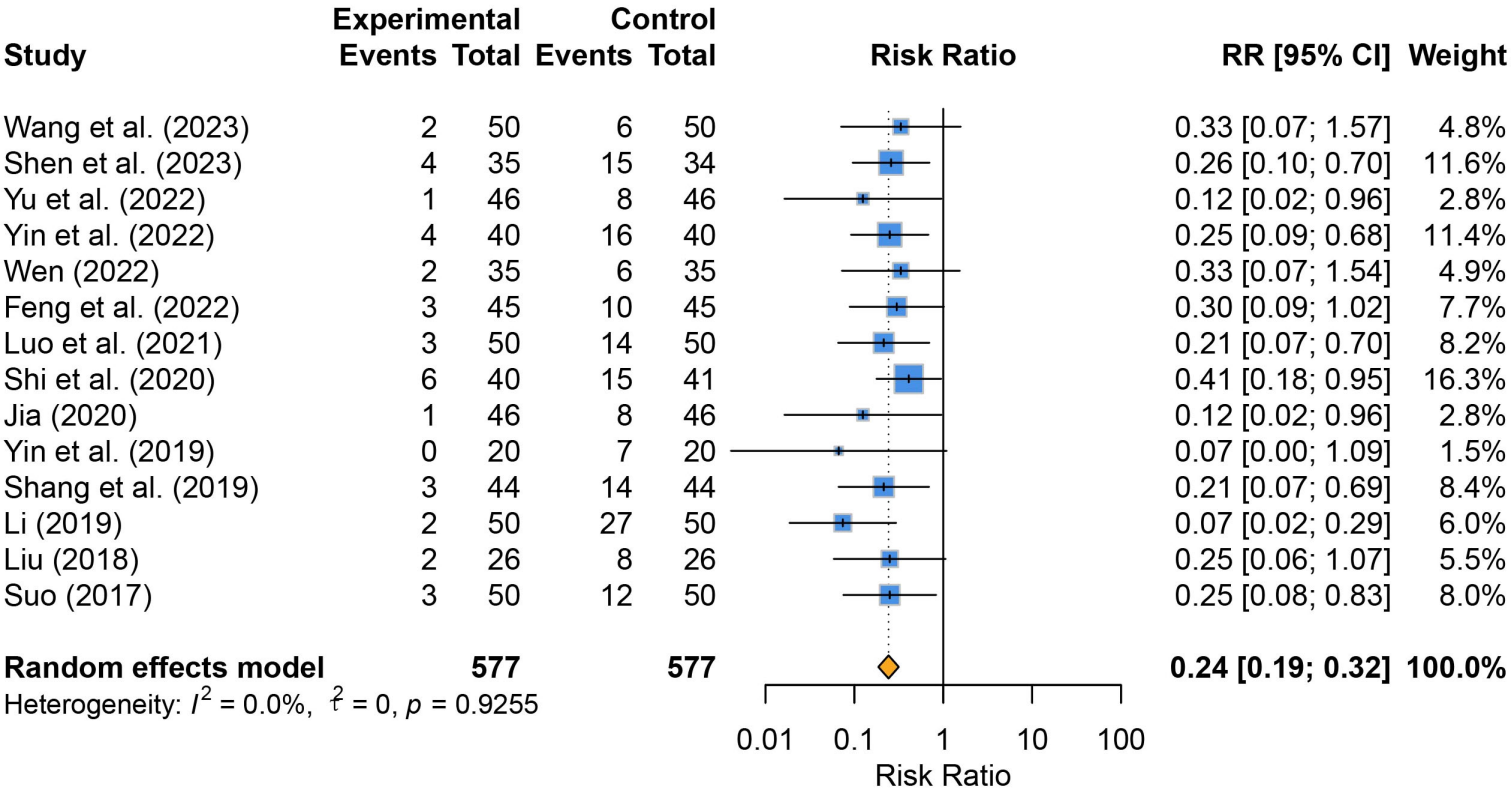
Forest plot of the meta-analysis on complication rates. This image presents the pooled effect of self-management interventions on the incidence of stoma-related complications in bladder cancer patients with a urinary ostomy.

The effect of self-management interventions on the incidence of stoma-related complications was analyzed using data from 14 studies involving 1,040 participants. The pooled analysis, presented in **Figure 13**, demonstrated that the intervention significantly reduced the risk of complications compared to routine care [Risk Ratio (RR) = 0.24, 95% CI: 0.19 to 0.32, *P* < 0.001]. This represents a 76% relative risk reduction. There was no statistically significant variation among the studies (I^2^ = 0.0%, *P* = 0.93), suggesting that the results from all of the included trials were consistent.

### Effects of self-management interventions on anxiety

**Figure 14:**
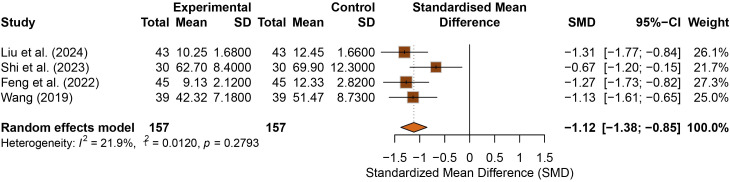
Forest plot of the meta-analysis on anxiety. This image displays the pooled effect of self-management interventions on anxiety levels in bladder cancer patients with a urinary ostomy.

Pooled analysis of 4 studies including 314 participants evaluated the impact of self-management interventions on anxiety levels. The findings, which are displayed in **Figure 14**, indicated a considerable effect size and a statistically significant decrease in anxiety favoring the intervention group (SMD = –1.12, 95% CI: –1.44 to –0.80; *P* < 0.001). Consistent results across trials were indicated by the low and non-statistically significant heterogeneity among studies (I2 = 21.9%, *P* = 0.28).

## Discussion

This systematic review and meta-analysis synthesized evidence from 27 randomized controlled trials involving bladder cancer patients with urinary ostomy to evaluate the effects of structured self-management interventions on self-care ability, QoL, psychological outcomes, and stoma-related complications. Our findings demonstrated that self-management interventions significantly improve self-care ability (SMD = 2.42, 95% CI: 1.63–3.22), enhance QoL (SMD = 1.44, 95% CI: 0.46–2.42), reduce anxiety (SMD = –1.12, 95% CI: –1.44 to –0.80), and markedly decrease the incidence of stoma-related complications (RR = 0.24, 95% CI: 0.19–0.32) compared with routine care. These results suggest that self-management interventions are effective strategies for promoting postoperative recovery and overall well-being in patients with urinary ostomy.

The observed improvements can be attributed to the multifaceted components of the interventions. Educational and skill-training elements, including preoperative stoma education, teach-back sessions, and hands-on practice, enhance patients’ knowledge and practical competencies, thereby increasing confidence in stoma management (17). By reinforcing learning through repeated practice and feedback, these components may reduce errors in daily stoma care and empower patients to manage complications proactively. Psychosocial support components, including motivational interviewing, peer support groups, and nurse–family–peer collaborative models, likely contribute to reductions in anxiety and improvements in psychological adaptation by addressing emotional needs and promoting social connectedness (18). These support mechanisms may also mitigate feelings of stigma, isolation, and loss of control that commonly accompany living with a urinary ostomy. Technology-assisted guidance, including WeChat-based follow-up and telehealth monitoring, facilitates continuous engagement, timely feedback, and adherence to self-care routines, which may explain the lower rates of stoma-related complications (19). These digital approaches extend care beyond hospital settings and allow early identification and management of emerging problems. Furthermore, interventions grounded in theoretical frameworks including Orem’s self-care theory, the Self-Transcendence Theory, and the IMB model provide structured guidance that enhances the effectiveness of self-management strategies (20). The use of theory-driven frameworks may improve intervention coherence and promote sustained behavioral change by clarifying mechanisms linking knowledge, motivation, and self-care behaviors. This interpretation is supported by the subgroup analyses, which showed that theory-driven interventions tended to produce greater improvements in QoL than comprehensive interventions without an explicit theoretical basis, suggesting that structured behavioral frameworks may facilitate more effective patient engagement and adaptation.

Our findings are broadly consistent with previous single-center RCTs and quasi-experimental studies that have reported improvements in self-care behaviors, QoL, and psychological outcomes following self-management interventions (13, 20). However, this meta-analysis extends the current literature by quantitatively pooling results across a larger sample, systematically evaluating both clinical (self-care ability, complications) and psychosocial outcomes (anxiety, QoL), and highlighting the overall magnitude of intervention effects. By integrating evidence across diverse intervention models and outcome measures, this study provides a more comprehensive assessment of the effectiveness of self-management interventions than individual trials alone. The consistency of the reduction in stoma-related complications across studies (I^2^ = 0%) further strengthens the evidence supporting the clinical benefit of self-management interventions. This finding suggests that, despite variability in intervention design, improving patients’ engagement in self-care consistently leads to better clinical outcomes. Moreover, subgroup analyses indicated that comprehensive interventions achieved the largest improvements in self-care ability, highlighting the potential value of combining education, skills training, psychosocial support, and follow-up management within an integrated intervention framework.

Nevertheless, substantial heterogeneity was observed for outcomes including self-care ability (I^2^ = 93.7%) and QoL (I^2^ = 96%), likely reflecting differences in intervention components, duration, frequency, theoretical frameworks, outcome measurement instruments, and patient characteristics. Although the interventions varied in their specific content and delivery formats, all shared a common core of structured education and skills training aimed at supporting daily self-management, which provided a conceptual basis for pooling the results. Subgroup and sensitivity analyses demonstrated a consistent direction of benefit across studies, supporting the overall robustness of the findings despite the observed heterogeneity. Additional subgroup analyses suggested that intervention duration and follow-up period may partly explain the variability in effect estimates. Larger improvements in self-care ability were generally observed in studies with shorter intervention durations and follow-up periods, whereas QoL benefits tended to be more evident with longer follow-up (21). These findings indicate that gains in self-management skills may occur relatively early after intervention, while broader improvements in quality of life may emerge more gradually over time. The wide prediction interval observed for QoL further suggests that the magnitude of benefit may vary across different clinical settings and patient populations (22).

Moreover, the methodological quality of the included trials warrants consideration when interpreting the pooled estimates. Most studies were rated as having some concerns regarding risk of bias, primarily due to insufficient reporting of randomization, allocation concealment, and blinding. These methodological limitations may systematically inflate treatment effects, particularly for patient-reported outcomes susceptible to detection bias. Consistent with the GRADE assessment, the certainty of evidence ranged from moderate to low across outcomes, reflecting concerns related to study limitations, heterogeneity, and publication bias. Therefore, although the overall findings consistently favored self-management interventions, the magnitude of some pooled effects should be interpreted with appropriate caution. Additionally, most included studies were conducted in China, with only one trial from Denmark, which may limit the generalizability of findings to other cultural and health care contexts. The effectiveness and implementation of self-management interventions may be influenced by differences in health care delivery models, availability of specialized stoma nursing services, patient education systems, and access to post-discharge support resources (23). Cultural factors may also affect patients’ health beliefs, self-care behaviors, family involvement, and willingness to engage in self-management activities, potentially influencing intervention uptake and outcomes (24). In addition, as this review included studies published in English and Chinese only, potential language bias cannot be excluded, although these two languages cover the vast majority of relevant clinical research in this field and are widely used in the reporting of ostomy-related studies. Therefore, caution is warranted when extrapolating the present findings to populations in other countries or regions with different health care infrastructures and sociocultural environments. Other limitations include moderate methodological quality in many trials, with common concerns related to inadequate reporting of randomization procedures, allocation concealment, and blinding, as well as the reliance on patient-reported outcomes. The predominance of short follow-up periods also restricts conclusions regarding the durability of intervention effects over time, particularly for long-term adaptation and complication prevention. Regarding the very large pooled effect size observed for self-care ability (SMD = 2.42), this finding may be partly explained by variations in baseline self-care levels and the relatively intensive, multifaceted nature of several interventions. In addition, small sample sizes in some included trials and limited reporting of methodological details may have contributed to an overestimation of the pooled effect. Publication bias was also suggested by the asymmetric funnel plot and significant Egger’s test results. Therefore, the magnitude of the pooled effect may be somewhat overestimated. These factors should be considered when interpreting the magnitude of the observed effect; nevertheless, the consistent direction of benefit across studies still supports a positive impact of self-management interventions on self-care ability.

Despite these limitations, the findings have important clinical and research implications. Clinically, structured self-management interventions that combine education, skills training, psychosocial support, and technology-assisted guidance should be considered integral to the postoperative care of bladder cancer patients with urinary ostomy. These interventions not only enhance patients’ self-care competence and QoL but also reduce complications and psychological distress. Integrating these interventions into routine nursing pathways may optimize resource utilization and support patient-centered care. For future research, high-quality, multicenter RCTs with standardized intervention protocols and validated outcome measures are needed to confirm the long-term effectiveness of self-management strategies and to explore the optimal combination of intervention components. Further studies should also examine cost-effectiveness and implementation feasibility in different health care systems. Additionally, cross-cultural studies could examine the generalizability of these interventions in diverse health care settings.

## Conclusion

This systematic review and meta-analysis indicates that structured self-management interventions can improve self-care ability, quality of life, psychological well-being, and stoma-related outcomes in bladder cancer patients with urinary ostomy. These findings support the integration of self-management interventions into routine postoperative care. Further high-quality multicenter trials with standardized protocols and longer follow-up are needed to confirm their long-term effectiveness.

## Data Availability

The original contributions presented in the study are included in the article/**Supplementary Material**. Further inquiries can be directed to the corresponding author.
