# Supplementary material for: Effects of self-management interventions on self-care ability and quality of life in patients with urinary ostomy after bladder cancer: a systematic review and meta-analysis
Source: Front Oncol. 2026 Jul 27;16:1864061. doi: 10.3389/fonc.2026.1864061 (PMC13453741; doi:10.3389/fonc.2026.1864061)
Supplement: Supplementary file 1 [file Table1.docx]

**Supplementary Table S1. Complete Search Strategies for All Databases**

| **Database** | **Search Strategy** |
| --- | --- |
| **PubMed** | ("Urinary Bladder Neoplasms"[Mesh] OR "Self-Management"[Mesh] OR "bladder cancer"[Title/Abstract] OR "bladder carcinoma"[Title/Abstract] OR "bladder tumor"[Title/Abstract] OR "bladder neoplasm"[Title/Abstract]) AND ("urinary ostomy"[Title/Abstract] OR "urostomy"[Title/Abstract] OR "ileal conduit"[Title/Abstract] OR "cutaneous ureterostomy"[Title/Abstract] OR "urinary diversion"[Title/Abstract] OR "urinary stoma"[Title/Abstract]) AND ("Self Care"[Mesh] OR "Patient Education as Topic"[Mesh] OR "Health Education"[Mesh] OR "self-management"[Title/Abstract] OR "self care"[Title/Abstract] OR "self-care"[Title/Abstract] OR "self efficacy"[Title/Abstract] OR "self-efficacy"[Title/Abstract] OR "health education"[Title/Abstract] OR "patient education"[Title/Abstract] OR "stoma care"[Title/Abstract] OR "nursing intervention"[Title/Abstract]) AND ("randomized controlled trial"[Publication Type] OR "controlled clinical trial"[Publication Type] OR "randomized"[Title/Abstract] OR "randomised"[Title/Abstract] OR "randomly"[Title/Abstract] OR "RCT"[Title/Abstract]) |
| **Cochrane Library** | #1 MeSH descriptor: [Urinary Bladder Neoplasms] explode all trees #2 (bladder cancer OR bladder carcinoma OR bladder tumor OR bladder neoplasm):ti,ab,kw #3 #1 OR #2 #4 (urinary ostomy OR urostomy OR ileal conduit OR cutaneous ureterostomy OR urinary diversion OR urinary stoma):ti,ab,kw #5 MeSH descriptor: [Self Care] explode all trees #6 MeSH descriptor: [Patient Education as Topic] explode all trees #7 MeSH descriptor: [Health Education] explode all trees #8 (self-management OR self care OR self-care OR self efficacy OR self-efficacy OR health education OR patient education OR stoma care OR nursing intervention):ti,ab,kw #9 #5 OR #6 OR #7 OR #8 #10 #3 AND #4 AND #9 |
| **Embase** | ('urinary bladder tumor'/exp OR 'bladder cancer':ti,ab OR 'bladder carcinoma':ti,ab OR 'bladder tumor':ti,ab OR 'bladder neoplasm':ti,ab) AND ('urostomy'/exp OR 'urinary ostomy':ti,ab OR 'urostomy':ti,ab OR 'ileal conduit':ti,ab OR 'cutaneous ureterostomy':ti,ab OR 'urinary diversion':ti,ab OR 'urinary stoma':ti,ab) AND ('self care'/exp OR 'patient education'/exp OR 'health education'/exp OR 'self-management':ti,ab OR 'self care':ti,ab OR 'self-care':ti,ab OR 'self efficacy':ti,ab OR 'self-efficacy':ti,ab OR 'health education':ti,ab OR 'patient education':ti,ab OR 'stoma care':ti,ab OR 'nursing intervention':ti,ab) AND ('randomized controlled trial'/exp OR 'controlled clinical trial'/exp OR 'randomized':ti,ab OR 'randomised':ti,ab OR 'randomly':ti,ab OR 'rct':ti,ab) |
| **CINAHL** | S1 (MH "Urinary Bladder Neoplasms+") OR TI ("bladder cancer" OR "bladder carcinoma" OR "bladder tumor" OR "bladder neoplasm") OR AB ("bladder cancer" OR "bladder carcinoma" OR "bladder tumor" OR "bladder neoplasm") S2 (MH "Urinary Diversion+") OR TI ("urinary ostomy" OR "urostomy" OR "ileal conduit" OR "cutaneous ureterostomy" OR "urinary diversion" OR "urinary stoma") OR AB ("urinary ostomy" OR "urostomy" OR "ileal conduit" OR "cutaneous ureterostomy" OR "urinary diversion" OR "urinary stoma") S3 (MH "Self Care+") OR (MH "Patient Education+") OR (MH "Health Education+") OR TI ("self-management" OR "self care" OR "self-care" OR "self efficacy" OR "self-efficacy" OR "health education" OR "patient education" OR "stoma care" OR "nursing intervention") OR AB ("self-management" OR "self care" OR "self-care" OR "self efficacy" OR "self-efficacy" OR "health education" OR "patient education" OR "stoma care" OR "nursing intervention") S4 (MH "Randomized Controlled Trials+") OR TI ("randomized" OR "randomised" OR "randomly" OR "RCT") OR AB ("randomized" OR "randomised" OR "randomly" OR "RCT") S5 S1 AND S2 AND S3 AND S4 |
| **Web of Science** | #1 TS=("bladder cancer" OR "bladder carcinoma" OR "bladder tumor" OR "bladder neoplasm") #2 TS=("urinary ostomy" OR "urostomy" OR "ileal conduit" OR "cutaneous ureterostomy" OR "urinary diversion" OR "urinary stoma") #3 TS=("self-management" OR "self care" OR "self-care" OR "self efficacy" OR "self-efficacy" OR "health education" OR "patient education" OR "stoma care" OR "nursing intervention") #4 TS=("randomized" OR "randomised" OR "randomly" OR "RCT" OR "randomized controlled trial") #5 #1 AND #2 AND #3 AND #4 |
| **CNKI** | 主题 = (膀胱癌 OR 膀胱肿瘤) AND 主题 = (尿路造口 OR 泌尿造口 OR 尿流改道 OR 回肠膀胱术 OR 输尿管皮肤造口) AND 主题 = (自我管理 OR 自我护理 OR 自我效能 OR 健康教育 OR 造口护理) AND 主题 = (随机 OR 随机对照试验 OR 随机分组 OR RCT) |
| **WanFang** | 主题:(膀胱癌 OR 膀胱肿瘤) AND 主题:(尿路造口 OR 泌尿造口 OR 尿流改道 OR 回肠膀胱术 OR 输尿管皮肤造口) AND 主题:(自我管理 OR 自我护理 OR 自我效能 OR 健康教育 OR 造口护理) AND 主题:(随机 OR 随机对照试验 OR 随机分组 OR RCT) |
| **VIP** | 主题=(膀胱癌 OR 膀胱肿瘤) AND 主题=(尿路造口 OR 泌尿造口 OR 尿流改道 OR 回肠膀胱术 OR 输尿管皮肤造口) AND 主题=(自我管理 OR 自我护理 OR 自我效能 OR 健康教育 OR 造口护理) AND 主题=(随机 OR 随机对照试验 OR 随机分组 OR RCT) |

**Supplementary Table S2. Certainty of evidence for self-management interventions vs routine care**

| **Certainty assessment** | | | | | | | **№ of patients** | | **Effect** | **Certainty** |
| --- | --- | --- | --- | --- | --- | --- | --- | --- | --- | --- |
| **№ of studies** | **Study design** | **Risk of bias** | **Inconsistency** | **Indirectness** | **Imprecision** | **Publication bias** | **Intervention** | **Control** | **SMD with 95% CI** |  |
| **Self-care ability** | | | | | | | | | | |
| **20** | RCT | Serious | Very serious | Not serious | Not serious | Serious | 635 | 622 | 2.42 (1.63, 3.22) | Low |
| **Quality of life** | | | | | | | | | | |
| **10** | RCT | Serious | Very serious | Not serious | Not serious | Undetected | 473 | 468 | 1.44 (0.46, 2.42) | Low |
| **Stoma-related complications** | | | | | | | | | | |
| **14** | RCT | Serious | Not serious | Not serious | Not serious | Undetected | 526 | 514 | 0.24 (0.19, 0.32) | Moderate |
| **Anxiety** | | | | | | | | | | |
| **4** | RCT | Serious | Not serious | Not serious | Serious | Undetected | 157 | 157 | −1.12 (−1.44, −0.80) | Low |
